# Efficacy of Resistance to *Francisella* Imparted by ITY/NRAMP/SLC11A1 Depends on Route of Infection

**DOI:** 10.3389/fimmu.2017.00206

**Published:** 2017-03-15

**Authors:** Daniel A. Powell, Jeffrey A. Frelinger

**Affiliations:** ^1^Department of Immunobiology, University of Arizona, Tucson, AZ, USA

**Keywords:** SLC11A1, natural resistance-associated macrophage protein, host response, *Francisella tularensis*, mouse genetics

## Abstract

Natural resistance-associated macrophage protein (NRAMP) encoded by the *Slc11a1* gene is a membrane-associated transporter of divalent metal ions. Murine *Slc11a1* has two known alleles, a functional *Slc11a1*^Gly169^, which is found in DBA2/J, NOD/LtJ, and 129p3/J and related mouse strains, and a non-functional *Slc11a1*^Asp169^, that is found in C56Bl/6J (B6) and BALB/cJ mice. B6 mice congenic for *Slc11a1*^Gly169^ (B6-*Slc11a1^G169^*) are markedly resistant to the intracellular pathogens *Salmonella, Leishmania*, and *Mycobacterium tuberculosis*. We examined the host cell response and replication of *Francisella* in B6-*Slc11a1^G169^* mice. Bone marrow-derived macrophages from either B6-*Slc11a1^G169^* or B6 mice were both effectively invaded by *Francisella* live vaccine strain (LVS). However, at 16 hours post-infection (hpi), the number of LVS bacteria recovered from B6 macrophages had increased roughly 100-fold, while in B6-*Slc11a1^G169^* mice the number decreased 10-fold. When the mice were challenged intranasally (i.n.) B6 mice lost significant amounts (~15%) of weight, where as B6-*Slc11a1^G169^* mice lost no weight. Three days after infection in B6-*Slc11a1^G169^* mice, we failed to recover viable *Francisella* from the lungs, livers, or spleens. By contrast, B6 mice had bacterial burdens approaching 1 × 10^6^ CFU/organ in all three organs. To further examine the degree of resistance imparted by *Slc11a1*^Gly169^ expression, we challenged mice deficient in TLR2, TLR4, and TLR9, but expressing the functional Slc11a1 (B6-*Slc11a1^G169^Tlr2/4/9^−/−^*). Surprisingly, B6-*Slc11a1^G169^Tlr2/4/9^−/−^* mice had no notable weight loss. Eighty percent of B6-*Slc11a1^G169^Tlr2/4/9^−^*^/^*^−^* mice yielded no detectable *Francisella* in any organ tested. Additionally, *Slc11a1*^G169^ produced little detectable cytokine either in the lung or serum compared to B6 mice. Mice expressing *Slc11a1*^Gly169^ survived even high doses (~80 LD_50_) of LVS inoculation. These data taken together serve to highlight that functional *Slc11a1*^Gly169^ can compensate the lack of TLR2/4/9. Thus *Slc11a1* is a critical player in murine resistance to pulmonary *Francisella* infection, but not footpad infection.

## Introduction

Many host genes have been implicated in the control of intracellular bacterial infections. The Slc11a gene was originally described as responsible for immunity to *Salmonella* (Ity) and has been found to function in many other intracellular bacterial infections including *Mycobacterium tuberculosis* (1–3). Common inbred mice carry one of two different alleles for Sc11a1. C57BL/6 (B6) and BALB/c the most commonly used inbred mouse strains in immunology carry a non-functional variant (G169D) allele (*Slc11a1^−^*). C3H and 129 (and other strains) carry the functional allele 169G (*Slc11a1^+^*).

The innate immune response to *Francisella tularensis* (Ft) has been the subject of investigation for the past decade. Ft is a Gram-negative, facultative intracellular pathogen. *In vivo* and *in vitro* Ft largely infects myeloid cells. Based on its role in other intracellular bacterial infections, we hypothesized that Slc11a1 would impact *Francisella* infection. However, nearly all reports of *Francisella* immunity have used either B6 or BALB/c mice, eliminating the ability to detect the impact of Slca11a on the infection (4). There is one early report that investigated the role of Slc11a1, and compared B10 mice and a congenic line B10.A-*slca11a1^G169^* following footpad challenge with *Francisella* strain live vaccine strain (LVS) (5). In that report they concluded that not only was a functional Slc11a not protective, but slightly increased susceptibility as measured by bacterial load in the spleen. However, no survival data were reported. One other report examined a sampling of inbred strains, including C3H and 129 that express functional Slca11a were not markedly more resistant to the most highly pathogenic *Francisella* strain, suggesting that Slc11a1 was not sufficient to mediate protection in that setting (6).

*Francisella* infection outcomes are markedly different depending on the route of infection. In our lab, we have demonstrated that the first cells infected, and the resulting immune response differs depending on the route of infection (7). Intranasal infection resulted in a high proportion of alveolar macrophages infected, whereas intradermal infection resulted in primarily neutrophils being infected. These changes resulted in a distinct adaptive immune response. Since *Slc11a1* is preferentially expressed in macrophages and alveolar macrophages, we speculated that the impact of Slc11a1 might differ depending on the route of infection.

Pathogen-associated molecular patterns are stimulators of innate immune response and play a role in *Francisella* infection. Mice defective in TLR2, but not TLR4 are more susceptible to *Francisella* infection (8). It is striking that these experiments were all performed in the absence of a functional *Slc11a1* gene. We speculated that Slc11a1 might compensate for a TLR2 defect. We tested TLR2-deficient mice with and without expression of Slc11a1^G169^ for their resistance to Ft LVS. Surprisingly, *Slc11a1^G169^* expressing mice were nearly equivalent to TLR2^+^/Slc11a1^G169^ mice indicating that Slc11a1^+^ can compensate for the lack of TLR2 expression.

## Materials and Methods

### Bacteria

*Francisella tularensis* subsp. *holarctica* LVS was obtained from the Centers for Disease Control (Atlanta, GA, USA). All bacterial studies were carried out with approval from the University of Arizona Biosafety Committee. Bacteria were grown at 37°C on chocolate agar supplemented with 1% IsoVitalex (Becton Dickinson). Inocula were prepared from lawn grown *Francisella*. Bacteria were resuspended in sterile phosphate-buffered saline (PBS) at an optical density at 600 nm (OD_600_) of 1, equivalent of 1 × 10^10^ CFU/mL. Innocula were diluted in sterile PBS to obtain the desired bacterial dose. Challenge doses were quantified by serial dilution and plating on chocolate agar.

### Bone Marrow-Derived Macrophage (BMDM) Generation

Primary BMDMs were cultured from femurs as previously described (9). Following differentiation, non-adherent cells were removed by multiple washes with PBS and BMDMs were removed from plates by scraping. Cells were cultured in Dulbecco’s modified Eagle’s medium (DMEM) supplemented with 10% fetal bovine serum (Atlas), l-glutamine (HyClone), sodium pyruvate (HyClone), and penicillin–streptomycin (Life Technologies). Medium was replaced with antibiotic-free medium 24 h prior to inoculation with *Francisella*.

### *Francisella* Growth Assays

A total of 2 × 10^5^ BMDM/well were seeded into 96-well flat bottom plates (~80% confluent) for *Francisella* intracellular growth assays and incubated 2 h to allow adherence to the plate. Cultures were inoculated with LVS at 1 or 100 bacteria per cell. Infection was facilitated by centrifugation at 300 × *g* for 5 min. Cells were incubated for 1 h with bacteria, and the medium was then removed. Fresh medium containing 50 μg/mL gentamicin (Sigma) was added to kill extracellular bacteria. One hour after gentamicin addition, medium was removed, and cells washed twice before the addition of fresh antibiotic-free medium. To determine intracellular growth, medium was removed at indicated time points post-infection, and 200 μL of PBS was added to the cultures. Cells were removed from the plate by vigorous pipetting. Cells were lysed by vortexing at maximal speed for 1 min. Serial 1:10 dilutions of the lysate were made and plated onto chocolate agar. The resulting colonies were counted 48 h later. For determination of reactive oxygen species (ROS), BMDM were harvested and processed with CellRox Green (ThermoFisher) per the manufacturers instructions.

### Mice

C57Bl/6J (B6) and B6.129-*Tlr2^tm1Kir^*/J (TLR2^−/−^) mice were obtained from The Jackson Laboratories (Bar Harbor, ME) and bred in-house. B6 *Slc11a1*^G169^ (*Slc11a1*^+^) and B6 *Slc11a1*^+^ *Tlr2/4/9^−^*^/^*^−^* (*Slc11a1*^+^ *Tlr2/4/9^−^*^/^*^−^*) were obtained from Dr. Gregory M. Barton (University of California, Berkley) and bred in-house. The B6-*Slc11a1^+^* mice are an incipient congenic, having been backcrossed to B6 five generations (10). This results in mice that are approximately 97% B6 background genes (excepting those linked to *Slc11a1*). The triple knockout has previously described and backcrossed to B6 (10). F1 mice (*Slc11a1*^±^ *Tlr*2*^−^*^/^*^−^Tlr*4^±^ *Tlr*9^±^) were produced by crossing *Slc11a1*^+^ *Tlr2/4/9^−^*^/^*^−^* with *Tlr*2*^−^*^/^*^−^*. Loss of TLR2 expression was confirmed by flow cytometry. All animal protocols were approved by The University of Arizona IACUC.

### Inoculation of Mice

For intranasal infection, mice were anesthetized with 575 mg/kg of body weight of tribromomethanol (Avertin; Sigma) administered intraperitoneally. Mice were then intranasally (i.n.) inoculated with various doses of LVS suspended in 25 μL PBS. For footpad infection, mice were restrained and inoculated with LVS suspended in 50 μL PBS in the right footpad. Mice were weighed daily following all inoculations. Mice were sacrificed if they lost more than 25% of their starting weight, as indicated in our University of Arizona IACUC protocol.

### Determination of Bacterial Burdens

Spleens, livers, and lungs were homogenized in sterile PBS by using a Biojector (Bioject) as previously described (11). Tenfold serial dilutions were plated onto chocolate agar. The resulting colonies were counted 72 h later. The limit of detection (LOD) was 50 CFU per organ.

### Preparation of Lung Cells

Lungs were perfused with PBS to remove blood and then finely minced. Minced lung was placed into 10 mL of digestion buffer containing 0.5 mg/mL collagenase I (Worthington Biochemical), 0.02 mg/mL DNase (Sigma), and 125 U/mL elastase (Worthington Biochemical) in RPMI 1640 (HyClone). Lungs were digested for 30 min at 37°C and then vigorously pipetted prior to filtering through a 100-μm filter. Red blood cells were lysed by using ammonium chloride–potassium carbonate lysis buffer. Viable cells were determined by trypan blue exclusion using a hemacytometer.

### Determination of Serum and Lung Cytokine Production

Serum and lung cytokines (IL-6, IL-10, MCP-1, IFN-γ, TNF-α, and IL-12p70) were determined using a cytometric bead array (CBA) Mouse Inflammation Kit (Becton Dickinson) according to the manufacturers instructions. For lungs, single cell suspensions were incubated in DMEM with antibiotics for 24 h and the supernatant was harvested and used for the CBA. Serum was analyzed undiluted.

### Antibodies and Flow Cytometry

The following directly conjugated antibodies were utilized for flow cytometry analysis: CD3 (clone 145-2C11; eBioscience), CD4 (clone GK1.5; Biolegend), CD8a (clone 53-6.7; BD Biosciences), CD11b (clone M1/70; Biolegend), CD11c (clone N418; Biolegend), CD19 (clone 6D5; Biolegend), F4/80 (clone BM8; Biolegend), and GR-1 (Ly-6G) (clone RB6-8C5; eBioscience). All antibodies were titrated on normal B6 splenocytes prior to use. Lung cells were prepared as above. After preparation of single cell suspensions, cells were blocked for 30 min at 4°C with 24G2 serum to block Fc receptors. Cells were then stained with indicated antibodies for 30 min at 4°C in the dark. Cells were then washed three times with PBS + 2%BSA and analyzed on a Becton Dickinson LSRII flow cytometer. FlowJo (Treestar) was used for all flow cytometry analysis.

## Results

### LVS Fails to Replicate in *Slc11a1*^+^ Macrophages

**Figure 1:**
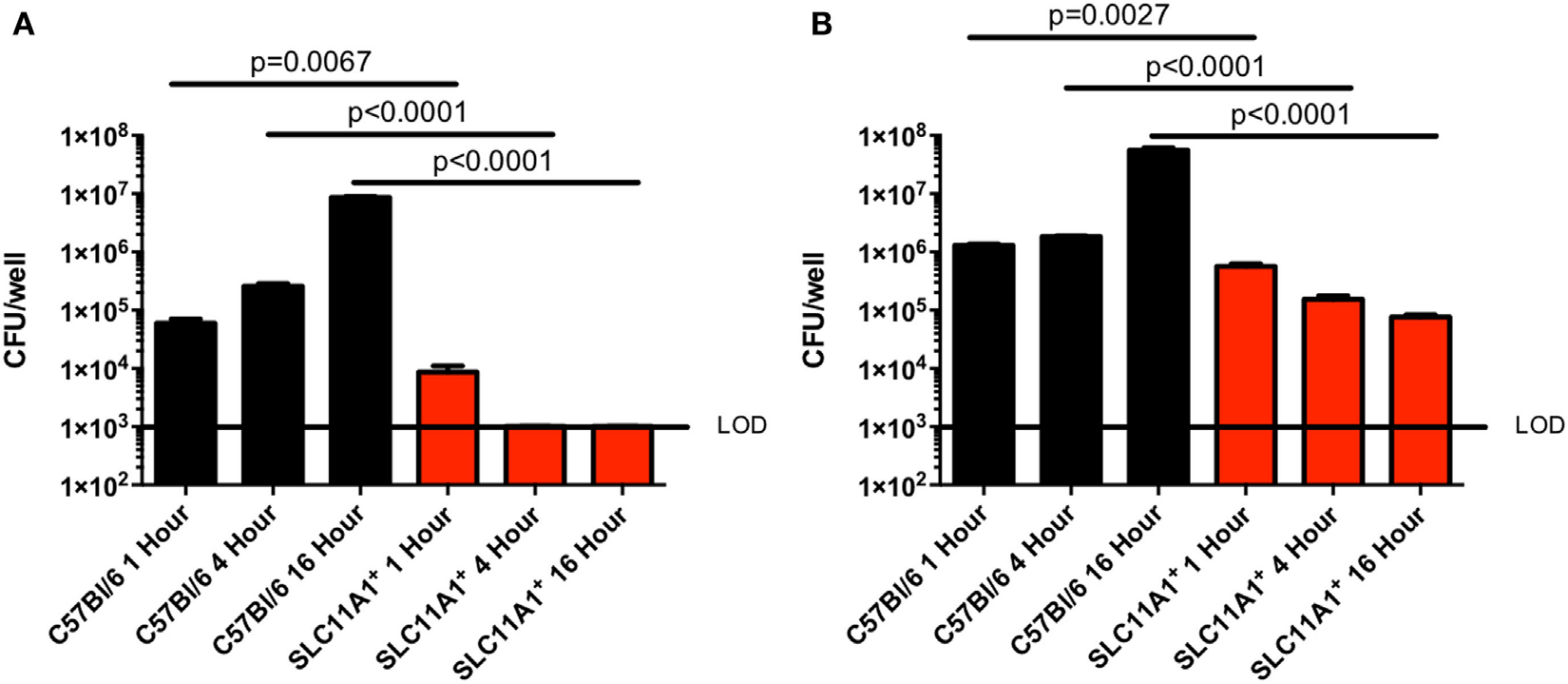
**Live vaccine strain (LVS) fails to replicate in *Slc11a1^+^* bone marrow-derived macrophage (BMDM)**. BMDMs from either B6 (black bars) mice or *Slc11a1^+^* (red bars) mice were infected with LVS at a multiplicity of infection of 1 **(A)** or 100 **(B)**. At the indicated times, macrophages were lysed and bacteria were enumerated by plating serial dilutions on chocolate agar. The solid line represents the limit of detection (LOD). A Student’s *t*-test on log-transformed data was used to calculate significance. *N* = 3 wells per time point, data are representative of three experiments of similar design. Error bars indicate SEM.

Since Slc11a1 has been previously implicated in control of intracellular bacterial infections and has only been superficially tested in *Francisella* infection, we tested the ability of LVS to replicate in BMDMs. We cultured BMDM from both B6 and *Slc11a1*^+^ mice and confirmed their macrophage phenotype (CD11b^+^ F4/80^+^) by flow cytometry. The BMDMs were then infected with LVS at either a multiplicity of infection (MOI) of 1 (Figure 1A) or 100 (Figure 1B). Two MOIs were used to ensure adequate infection. At 1, 4, and 16 hours post-infection (hpi), macrophages were lysed and viable bacterial enumerated. One hour after infection there were slightly fewer LVS in *Slc11a1*^+^ than B6 BMDM at both 100 and 1 MOI. By 4 h, there was a decrease in the number of LVS recovered from the *Slc11a1*^+^ BMDM at an MOI of 100, at the MOI of 1 there was no detectable LVS at 4 h in *Slc11a1*^+^ BMDM. The B6 BMDM had little change in burden at MOI of 100 and limited growth at the MOI of 1 at 4 hpi. By 16 h, LVS was still undetectable in *Slc11a1*^+^ BMDM at an MOI of 1, at the MOI of 100 there was a 100-fold decrease in bacteria recovered compared to the B6 BMDM and 10-fold compared to the *Slc11a1*^+^ 1 h after infection (Figure 1). Taken together these data indicate that LVS is slightly impaired in its ability to invade *Slc11a1^+^* BMDM, but more importantly is unable to proliferate after invasion.

### *Francisella* LVS Replicates to Similar Levels in following Foot Pad Inoculation in *Slc11a1*^+^ and *Slc11a1*^−^ Mice

**Figure 2:**
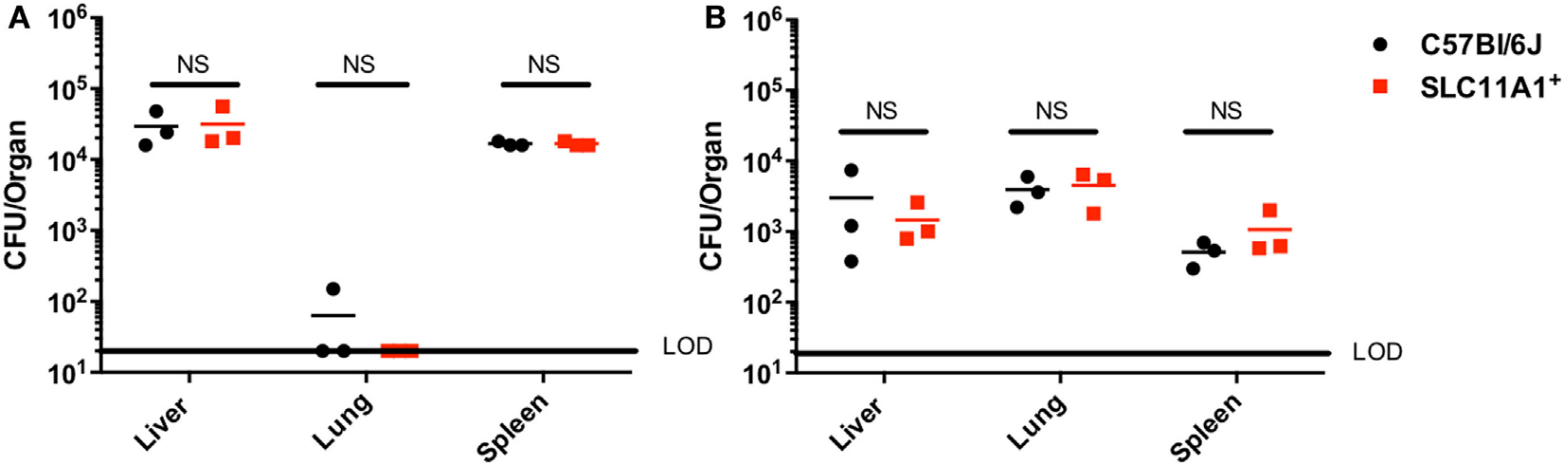
***Slc11a1^+^* mice show no difference compared to B6 in footpad infections with live vaccine strain (LVS)**. B6 (black circles) or *Slc11a1^+^* (red squares) mice were infected with 1,100 CFU of LVS *via* the footpad. At day 3 **(A)** or 5 **(B)** post-infection organs were harvested and bacterial burdens were enumerated by plating serial dilutions on chocolate agar. The solid line represents the limit of detection (LOD). A Student’s *t*-test on log-transformed data was used to calculate significance. No significant differences were found. *N* = 3 mice per time point, data are representative of two independent experiments of similar design.

Our *in vitro* experiments seemed to conflict with the earlier report that Slc11a1 increased susceptibility to LVS infection *in vivo* (5). Those experiments used sublethal footpad infection. The report of Kovarova used different mouse strains and we needed to test their route of infection with our strains. We therefore examined the effect of *Slc11a1* expression on footpad infection. *Slc11a1*^+^ and B6 mice were inoculated with a sublethal dose of 1,100 CFU of LVS. On days 3 and 7 post-infection, these mice were sacrificed and total bacterial burden was determined for the lung, liver, and spleen. In contrast to the previous report (5) where there was increased bacterial load in *Slc11a1*^+^ mice, we observed no significant difference in the burdens between *Slc11a1*^+^ and B6 mice in any organ tested at either time point (Figure 2).

### *Slc11a1*^+^ Mice Are Resistant to Intranasal LVS

**Figure 3:**
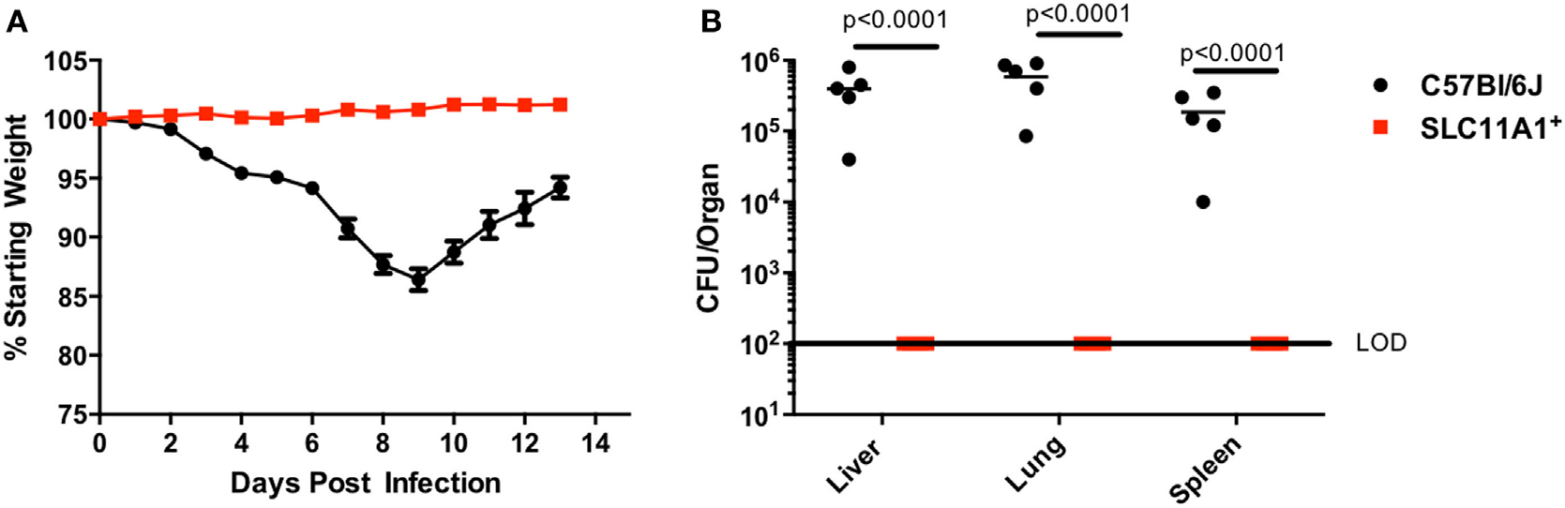
***Slc11a1^+^*mice show no weight loss or detectable bacteria after intranasal live vaccine strain (LVS) inoculation**. B6 (black circles) or *Slc11a1^+^* (red squares) mice were infected with 1,300 CFU of LVS *via* the intranasal route. **(A)** Mice were weighed daily as an indication of disease progression. **(B)** At 3 days post-infection organs were harvested from five mice and bacterial burdens were enumerated by plating serial dilutions on chocolate agar. The solid line represents the limit of detection (LOD). A Student’s *t*-test on log-transformed data was used to calculate significance. *N* = 5 mice per time point, data are representative of three experiments of similar design.

Since respiratory infection with *Francisella* alters the immune response compared to cutaneous infection, we sought to determine the role of Slc11a1 in intranasal *Francisella* infection (7). *Slc11a1*^+^ and B6 mice were infected with ~1,300 CFU LVS by the intranasal route. Mice were monitored for weight loss and survival and a subset were sacrificed at day 3 post-infection to determine organ burdens. Surprisingly, *Slc11a1*^+^ mice did not lose weight compared to their starting weight (Figure 3A). By contrast, B6 mice lost weight starting at day 3. At day 7, 2/5 surviving B6 mice had lost greater than 25% of their starting weight and were euthanized as required by our IACUC protocol. The mice who survived started regaining weight around day 10 (Figure 3A). In addition to maintaining weight, we were not able to recover any viable LVS from *Slc11a1*^+^ mice (LOD, 50 CFU) in any of the organs tested at day 3 post-infection. We recovered high levels of *Francisella* (10^5^–10^6^ CFU) from every organ tested in B6 mice (Figure 3B). These data demonstrate that Slc11a1 plays an important role in the resistance of mice to intranasal LVS infection. This is relevant to our previous results that demonstrated important differences in immune responses between intradermal and intranasal infection (7).

### *Slc11a1* Expression Can Compensate for Lack of TLR Expression

**Figure 4:**
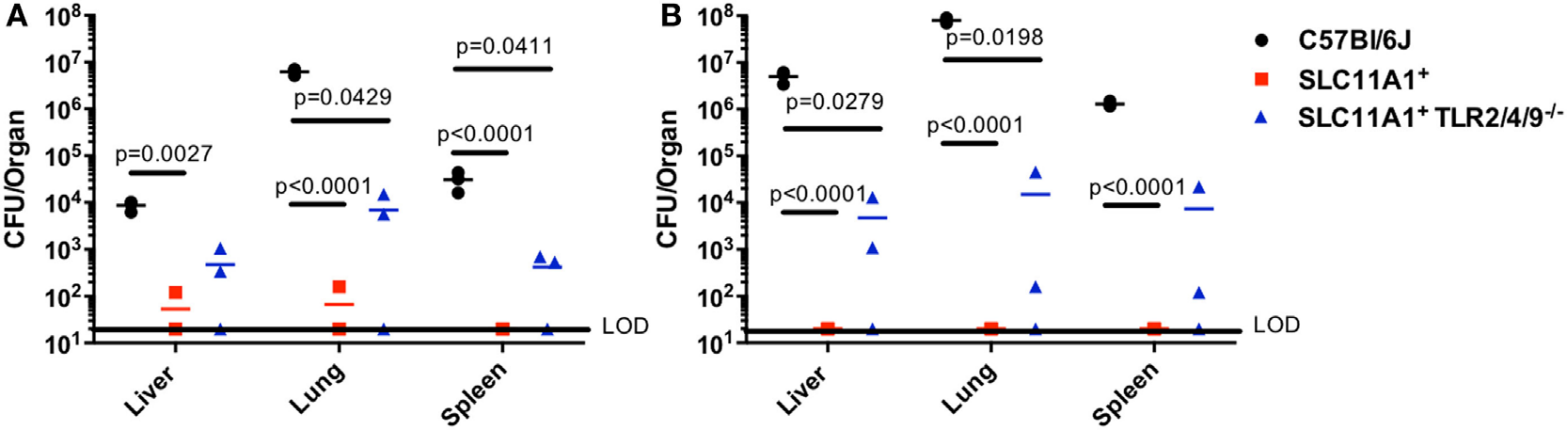
**Fewer bacteria are recovered from *Slc11a1^+^ Tlr2/4/9*^−^*^/^*^−^ mice than B6 mice following intranasal live vaccine strain (LVS) inoculation**. C57Bl/6 (black circles), *Slc11a1^+^* (red squares), or *Slc11a1^+^ Tlr2/4/9^−/−^* (blue triangles) mice were intranasally infected with 1,300 CFU of LVS. At 3 **(A)** and 5 **(B)** days post-infection organs were harvested and bacterial burdens were enumerated by plating serial dilutions on chocolate agar. The solid line represents the limit of detection (LOD). A Student’s *t*-test on log-transformed data corrected for multiple comparisons was used to calculate significance. Unless indicated, differences are not significant. *N* = 3 mice per time point, data are representative of two experiments of similar design.

TLR2 has been reported to play a critical role in Ft resistance. TLR2-deficient mice are more sensitive to Ft infection that B6 mice (8, 12). Since SLC11A1^+^ B6 mice were resistant to intranasal LVS infection, we wanted to determine if Slc11a1 expression could compensate for the loss of TLR2. We reasoned that *Slc11a1*^+^ *Tlr2/4/9^−^*^/^*^−^* mice would be at least as sensitive as TLR2 KO mice, as TLR2 has been strongly linked to resistance to Ft infection. We challenged *Slc11a1*^+^ *Tlr2/4/9^−^*^/^*^−^* mice along with *Slc11a1*^+^ and B6 mice with ~1,300 CFU of LVS i.n. as above. Similar to earlier experiments (Figure 3), most *Slc11a1*^+^ mice yielded no detectable LVS in the organs (LOD, 20 CFU) while we recovered mice high levels of bacteria on day 3 that increased into day 5 from B6 mice (Figures 4A,B). Surprisingly, *Slc11a1*^+^ *Tlr2/4/9^−^*^/^*^−^*, like *Slc11a1*^+^ mice, yielded 1,000-fold lower organ burdens than B6 in all organs tested, in spite of lacking TLR2/4/9 (Figures 4A,B). TLR4 has little role in *Francisella* pathogenesis as its unique LPS structure fails to bind TLR4. While EF-Tu and DNAK have been identified as ligands for TLR4, TLR4-deficient mice show no increase in sensitivity *Francisella* infection compared to B6 mice (13–16). TLR2 has been previously demonstrated to be important in survival of LVS infection in B6 genetic background mice. These experiments demonstrate that the presence of functional Slc11a1 can compensate for the TLR2 defect in resistance to Ft infection, although they are higher than *Tlr2*^+^ *Slc11a1*^+^ mice.

### *Slc11a1*^+^ Mice Produce Little Cytokine Compared to B6 Mice following Intranasal LVS Inoculation

**Figure 5:**
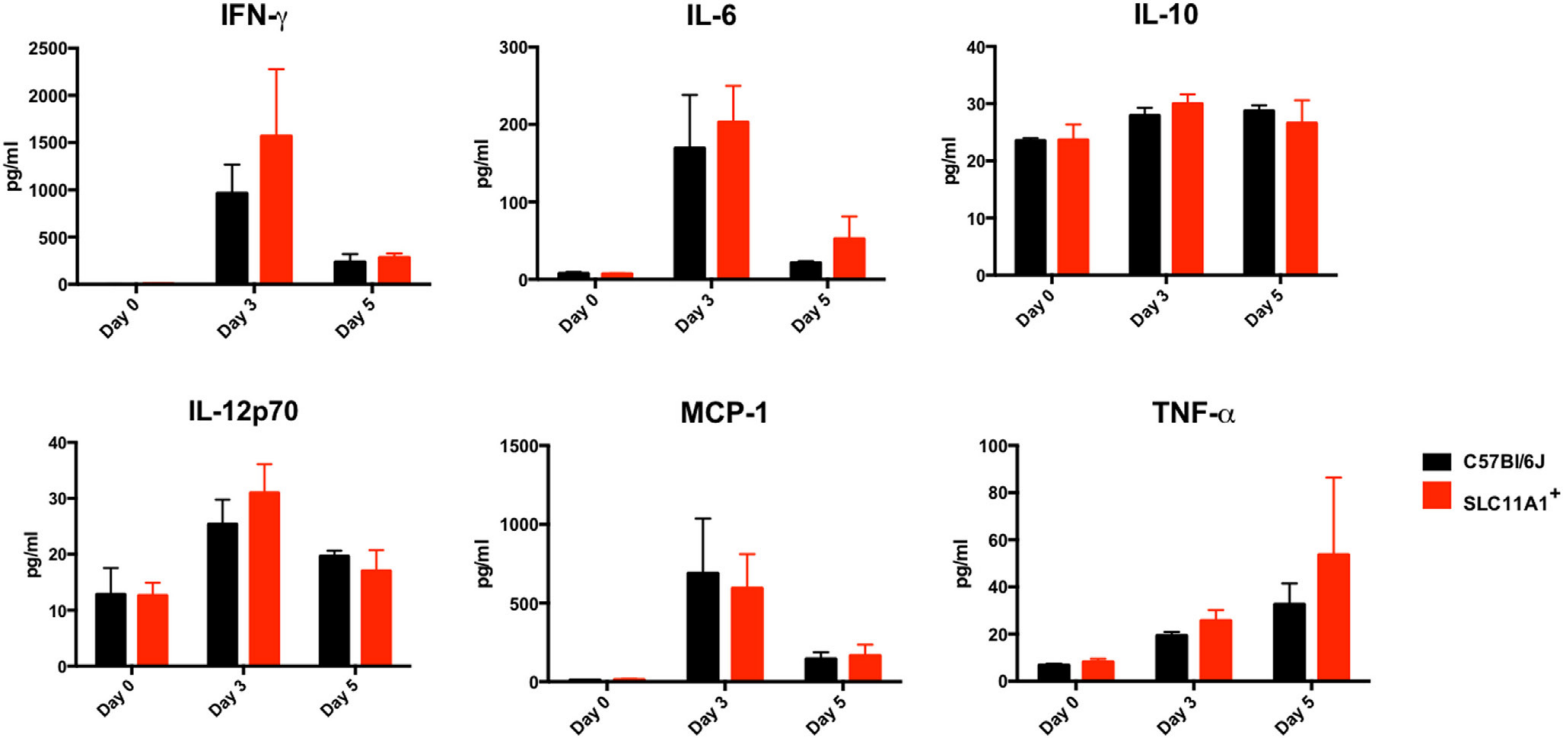
***Slc11a1^+^* and B6 mice produce similar cytokine following live vaccine strain (LVS) inoculation in the footpad**. B6 (black bars) or *Slc11a1*^+^ (red bars) mice were inoculated with 1,300 or 1,100 CFU of LVS in the footpad. Serum was harvested after 3 and 5 days, and cytokine was quantified by cytometric bead array. A Student’s *t*-test was used to calculate significance. Unless indicated, differences are not significant. *N* = 3 mice per time point, data are representative of two independent experiments of similar design.

**Figure 6:**
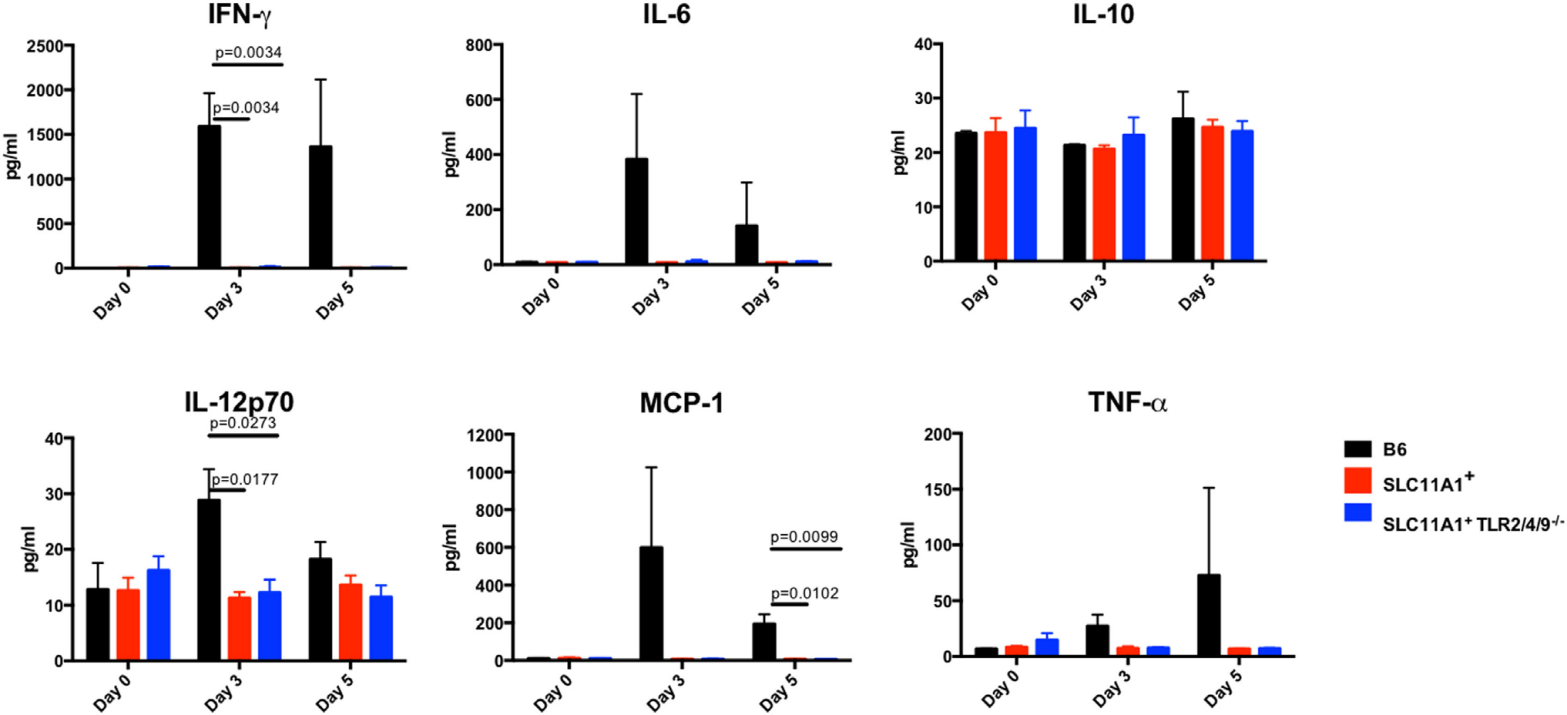
***Slc11a1^+^* mice show little detectable changes in serum cytokines after live vaccine strain (LVS) intranasal inoculation**. C57Bl/6 (black bars), *Slc11a1^+^* (red bars), or *Slc11a1^+^ Tlr2/4/9^−/−^* (blue bars) mice were infected with 1,300 CFU of LVS intranasally. Serum was harvested and cytokines were quantified by cytometric bead array. A Student’s *t*-test corrected for multiple comparisons was used to calculate significance. Unless indicated, differences are not significant. *N* = 3 mice per time point, data are representative of two independent experiments of similar design.

**Figure 7:**
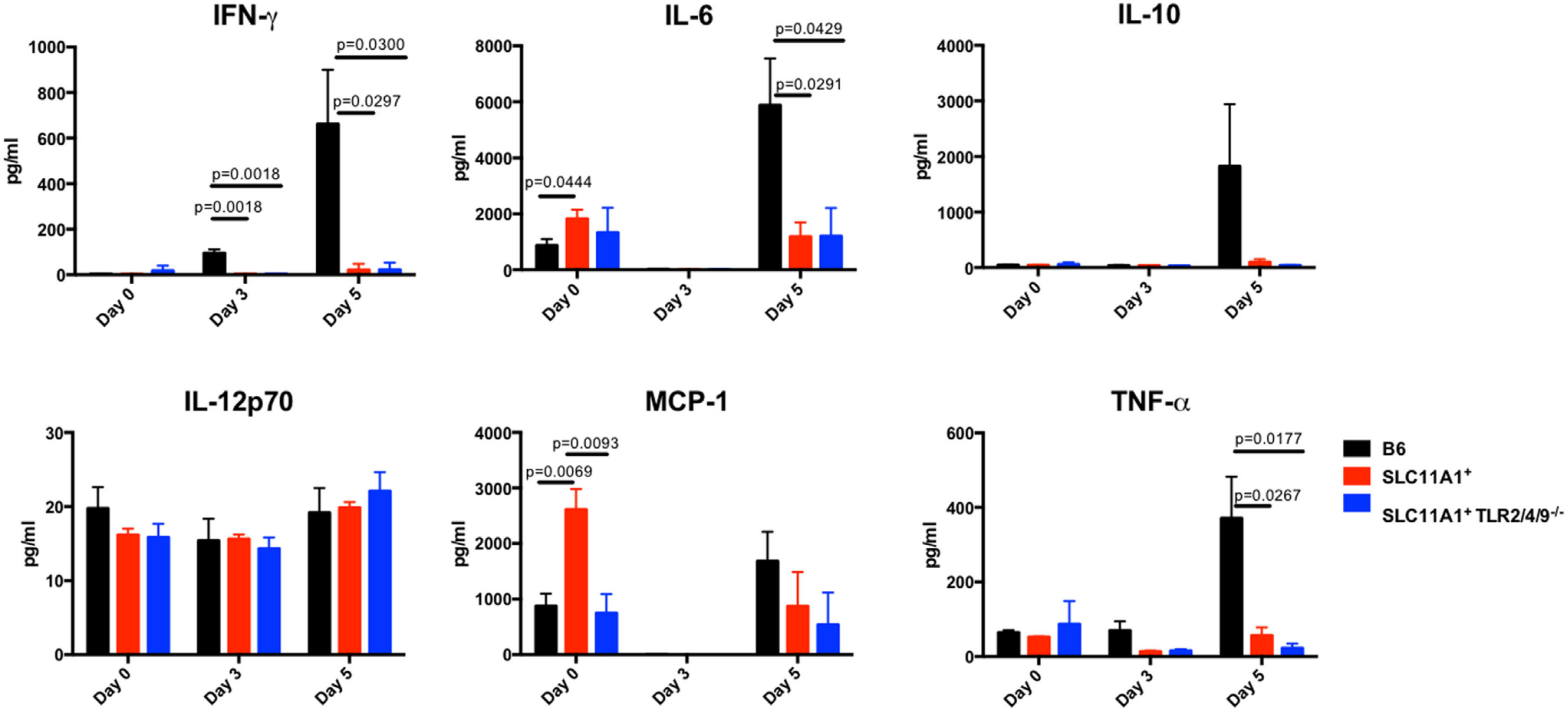
***Slc11a1^+^* mice show little detectable changes in lung cytokine after live vaccine strain (LVS) intranasal infection**. B6 (black bars), *Slc11a1^+^* (red bars), or *Slc11a1^+^ Tlr2/4/9^−/−^* (blue bars) mice were inoculated with 1,300 CFU of LVS *via* the intranasal route. At the indicated time point, lungs were harvested and processed into a single cellular suspension. After 24 h incubation, supernatants were harvested and cytokines were quantified by cytometric bead array. A Student’s *t*-test corrected for multiple comparisons was used to calculate significance. Unless indicated, differences are not significant. *N* = 3 mice per time point, data are representative of two independent experiments of similar design.

To examine the impact of functional *Slc11a1* expression on the innate immune response to LVS infection, we measured cytokine production in both the serum and lung homogenates of Ft infected mice. B6, *Slc11a1*^+^, and *Slc11a1*^+^ *Tlr2/4/9^−^*^/^*^−^* were i.n. infected with 1,300 CFU of LVS. Additional B6 and *Slc11a1*^+^ mice were infected with 1,000 CFU of LVS by the footpad route and serum cytokines measured. As a negative control, naïve mice received PBS by either the intranasal or footpad route. On day 3 and 5 post-infection, mice were sacrificed and cytokine production determined. Following the footpad infection, there were no significant differences between the B6 and *Slc11a1*^+^ mice (Figure 5). Both mouse strains produced high levels of IFN-γ, MCP-1, and IL-6. This is not surprising since they had similar LVS organ burdens in previous experiments (Figure 2). By contrast, following intranasal infection, the *Slc11a1*^+^ and *Slc11a1*^+^ *Tlr2/4/9^−^*^/^*^−^* mice accumulate very little cytokine in either in serum (Figure 6) or lung (Figure 7) after intranasal infection. B6 mice had significantly increased levels of IFN-γ and elevated levels of MCP-1, and IL-6 in the serum compared to *Slc11a1*^+^ and *Slc11a1*^+^ *Tlr2/4/9^−^*^/^*^−^* mice (Figure 6). Lung cultures from B6 mice produced significantly more IFN-γ, MCP-1, and IL-6 compared to *Slc11a1*^+^ and *Slc11a1*^+^ *Tlr2/4/9^−^*^/^*^−^* cultures (Figure 7). The lack of cytokine response seen in *Slc11a1*^+^ mice demonstrates it is not an increased innate cytokine response that protects *Slc11a1*^+^ mice and mediates bacterial clearance.

### B6 Mice Have Increased Lung Cellularity after Intranasal Infection Driven by Neutrophil Recruitment

Because we found so little cytokine in *Slc11a1*^+^ mice we hypothesized the cellular milieu in the lung would differ after infection. To determine the total cellularity of lungs B6, *Slc11a1*^+^, and *Slc11a1*^+^ *Tlr2/4/9^−^*^/^*^−^* were i.n. infected with 1,300 CFU of LVS, naïve mice received PBS as a control. At day 3 and 5 post-infection, mice were sacrificed and lungs were harvested, the right lobe was used for CFU determination (Figure 4) and the left lobe was used for lung cellularity counts and flow cytometry (Table 1). The naïve mice had no significant changes in total lung cellularity among strains. Consistent with previously published data, B6 mice demonstrated a significant increase in total lung cellularity at both days 3 and 5 (17). Strikingly in *Slc11a1*^+^ mice, we detected no increased cellularity compared to naïve mice at either 3 or 5 days post-infection. The lack of cellular infiltrate in the *Slc11a1*^+^ is striking, but consistent with the low levels of bacteria present. We detected only a moderate increase of cellularity in the *Slc11a1*^+^ *Tlr2/4/9^−^*^/^*^−^* mice at day 3 although still significantly lower than the B6 mice. By day 5, the cellularity of all *Slc11a1*^+^ *Tlr2/4/9^−^*^/^*^−^* mice had decreased close to naïve levels although B6 levels remained high (Table 1).

**Table 1:** **Cell subtypes in lung after live vaccine strain infection**.

	C57Bl/6	SCL11A1^+^	TLR2/4/9^−/−^ SLC11A1^+^	C57Bl/6	SLC11A1^+^	TLR2/4/9^−/−^ SLC11A1^+^	C57Bl/6	SLC11A1^+^	TLR2/4/9^−/−^ SLC11A1^+^

	Day 0	Day 0	Day 0	Day 3	Day 3	Day 3	Day 5	Day 5	Day 5
Total cells/lobe	3.40 × 10^6^ ± 2.08 × 10^5^	3.50 × 10^6^ ± 1.73 × 10^5^	3.50 × 10^6^ ± 1.73 × 10^5^	6.77 × 10^6^ ± 2.60 × 10^5^	3.53 × 10^6^ ± 6.67 × 10^4^	5.33 × 10^6^ ± 6.67 × 10^4^	7.63 × 10^6^ ± 2.61 × 10^5^	3.67 × 10^6^ ± 2.60 × 10^5^	4.40 × 10^6^ ± 2.60 × 10^5^
		NS	NS		*p* = 0.0006	*p* = 0.0120		*p* = 0.0002	*p* = 0.0006

Neutrophils (CD3^−^, CD19^−^, CD11c^Var^, CD11b^+^, Gr-1^+^)	9.79 × 10^4^ ± 1.06 × 10^4^	8.64 × 10^4^ ± 1.11 × 10^4^	7.77 × 10^4^ ± 2.33 × 10^4^	1.23 × 10^6^ ± 3.49 × 10^5^	2.45 × 10^5^ ± 2.03 × 10^4^	4.13 × 10^5^ ± 9.88 × 10^4^	7.63 × 10^6^ ± 2.60 × 10^5^	2.30 × 10^5^ ± 5.57 × 10^4^	4.24 × 10^5^ ± 2.00 × 10^4^
		NS	NS		NS	NS		*p* < 0.0001	*p* < 0.0001

Macrophages (CD3^−^, CD19^−^, CD11c^Var^, CD11b^+^, Gr-1^−^ F4/80^low^)	8.91 × 10^4^ ± 6.28 × 10^3^	8.57 × 10^4^ ± 2.99 × 10^3^	9.32 × 10^4^ ± 8.42 × 10^3^	4.48 × 10^5^ ± 7.26 × 10^4^	1.78 × 10^5^ ± 1.93 × 10^4^	2.35 × 10^5^ ± 8.24 × 10^3^	6.37 × 10^5^ ± 2.14 × 10^5^	1.79 × 10^5^ ± 4.79 × 10^4^	1.51 × 10^5^ ± 1.50 × 10^4^
		NS	NS		*p* = 0.046	NS		NS	NS

Macrophages (CD3^−^, CD19^−^, CD11c^Var^, CD11b^+^, Gr-1^−^ F4/80^mid^)	8.75 × 10^5^ ± 4.74 × 10^3^	8.07 × 10^5^ ± 1.57 × 10^4^	5.15 × 10^5^ ± 8.39 × 10^3^	1.41 × 10^5^ ± 8.44 × 10^4^	6.90 × 10^4^ ± 4.09 × 10^3^	1.48 × 10^5^ ± 3.06 × 10^3^	7.07 × 10^4^ ± 1.66 × 10^4^	6.11 × 10^4^ ± 1.53 × 10^4^	9.35 × 10^4^ ± 3.21 × 10^4^
		NS	NS		NS	NS		NS	NS

Dendritic cells (CD3^−^, CD19^−^, CD11c^+^, CD11b^−^, F4/80^low^)	1.21 × 10^4^ ± 2.44 × 10^2^	1.41 × 10^4^ ± 1.13 × 10^3^	1.60 × 10^4^ ± 4.39 × 10^3^	4.59 × 10^4^ ± 1.42 × 10^4^	1.71 × 10^4^ ± 8.83 × 10^2^	2.62 × 10^4^ ± 7.49 × 10^2^	5.11 × 10^4^ ± 2.05 × 10^4^	2.03 × 10^4^ ± 1.85 × 10^3^	2.45 × 10^4^ ± 4.09 × 10^3^
		NS	NS		NS	NS		NS	NS

Alveolar macrophages (CD3^−^, CD19^−^, CD11c^+^, CD11b^−^, F4/80^mid^)	5.22 × 10^4^ ± 3.66 × 10^3^	5.25 × 10^4^ ± 1.00 × 10^4^	4.37 × 10^4^ ± 9.84 × 10^3^	1.15 × 10^5^ ± 2.68 × 10^4^	9.70 × 10^4^ ± 1.45 × 10^3^	2.36 × 10^5^ ± 6.461 × 10^3^	3.22 × 10^4^ ± 6.01 × 10^3^	4.76 × 10^4^ ± 3.79 × 10^3^	9.97 × 10^4^ ± 1.90 × 10^4^
		NS	NS		NS	p = 0.0234		NS	NS

*Summary of total number of each cell type in the lungs of infected mice. Significant differences were made by comparing each strain to B6 at that time point (NS = not significant). A Student’s *t*-test corrected for multiple comparisons was used to calculate significance. *N* = 3 mice per time point*.

To determine which cells are responsible for this increased cellularity, samples were stained for flow cytometric analysis. The gating strategy for this analysis is in Figure S1 in Supplementary Material. Both the total number and the percentage of macrophages, dendritic cells, neutrophils, T, and B lymphocytes were determined. In B6 mice, two cell types were significantly increased, in both number and percentage. Neutrophils (CD3^−^, CD19^−^, CD11b^+^, Gr-1^+^) and monocyte/macrophages (CD3^−^, CD19^−^, CD11b^+^, Gr-1^−^, F4/80^−^) (Table 1) were increased. There was no significant difference in any other population in either *Slc11a1*^+^ or *Slc11a1*^+^ *Tlr2/4/9^−^*^/^*^−^* mice. This is consistent with Figure 1 showing the inability of *Francisella* to replicate in the myeloid cells of *Slc11a1*^+^ mice. Thus, the resistance to infection is not due to increased recruitment of innate cells, but rather a cell intrinsic property of the resident infected cells of *Slc11a1*^+^ mice.

### Following LVS Infection *Slc11a1*^+^ Mice Produce More ROS than B6 Mice

**Figure 8:**
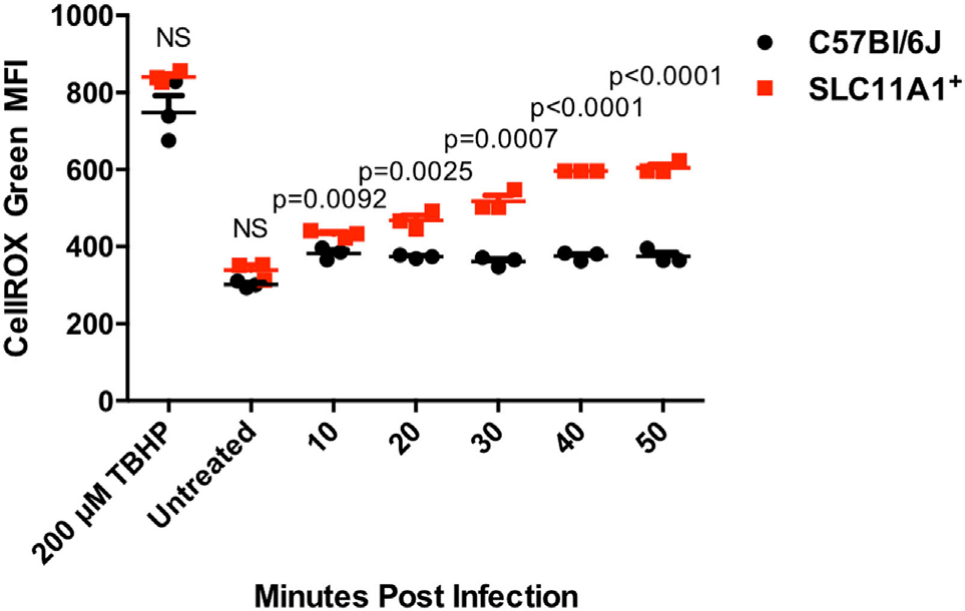
***Slc11a1^+^* bone marrow-derived macrophage (BMDM) show increased reactive oxygen species (ROS) compared to B6 in response to live vaccine strain (LVS) infection**. BMDMs were generated from either B6 (black circles) mice or *Slc11a1^+^* (red circles) mice and infected with LVS at a multiplicity of infection 10. Control cells were treated for 50 min with 200μM tert-butyl hyperoxide (TBHP). At the indicated time point, macrophages were harvested and processed for determination of ROS by CellROX green on the flow cytometer. A Student’s *t*-test corrected for multiple comparisons was used to calculate significance. *N* = 3 wells per time point. Data are representative of two independent experiments of similar design.

One mechanism used by *Francisella* for controlling the innate immune response is the reduction of host produced ROS (18, 19). To examine the ability of *Slc11a1*^+^ cells to produce ROS in response to LVS infection, BMDMs were cultured from B6 and *Slc11a1*^+^ mice and infected. We measured ROS production every 10 min. Control cells were treated with 70% tert-butyl hydroperoxide as a positive control or left untreated. Control cultures were harvested at 50 min after the infection. Both untreated B6 and *Slc11a1*^+^ BMDM produced similar low levels of ROS. In response to the positive control, tert-butyl hydroperoxide, the *Slc11a1*^+^ BMDM made significantly more ROS than the B6 cells. As seen previously, the B6 cells made little ROS in response to LVS infection. Strikingly, the *Slc11a1*^+^ BMDM made significantly more ROS in response to LVS starting at 20 min after infection and increasing throughout the assay (Figure 8). Thus the expression of functional Slc11a1 overrides the inhibition of ROS production by *Francisella* presumably mediating better early pathogen control both *in vitro* and *in vivo*.

### *Slc11a1*^+^ Mice Are Resistant to High-Dose Intranasal LVS Challenge

**Figure 9:**
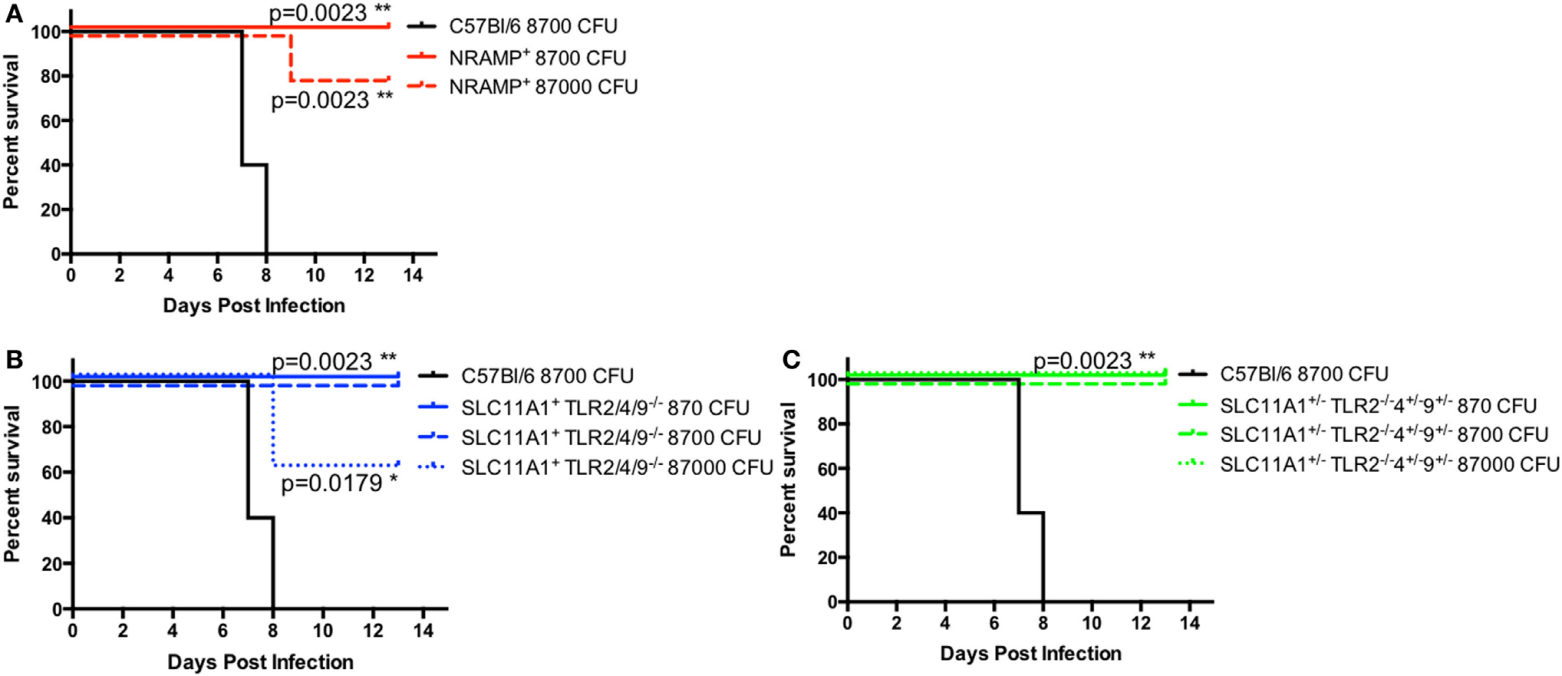
***Slc11a1^+^* mice are resistant to high-dose challenges of live vaccine strain (LVS) by the intranasal route**. B6 [black lines **(A–C)**], *Slc11a1^+^* [red lines **(A)**], *Slc11a1^+^ Tlr2/4/9^−/−^* [blue lines **(B)**], or *Slc11a1^±^ Tlr2^−/−^/4^±^/9^±^* [green lines **(C)**] mice were infected with the indicated doses of LVS *via* the intranasal route. Mice were monitored for survival and signs of disease. Significance was determined using a Mantel–Cox log rank test. *N* = 5 mice per group.

Because of the low bacterial proliferation in *Slc11a1*^+^ mice, we hypothesized that *Slc11a1*^+^ mice would be resistant to high-dose LVS challenge. In order to determine the extent of protection conferred by a functional Slc11a1—B6, *Slc11a1*^+^, *Slc11a1*^+^ *Tlr2/4/9^−^*^/^*^−^*, and *Slc11a1*^±^ *Tlr2^−^*^/^*^−^Tlr4*^±^ *Tlr9*^±^ mice were challenged i.n. with increasing doses of LVS and monitored for survival. B6 mice only received 8,700 CFU of LVS (~8 LD_50_). All B6 mice succumbed to infection by day 8 as expected. *Slc11a1*^+^ mice received either 8,700 CFU (~8 LD_50_) or 87,000 (~80 LD_50_) of LVS. All mice (5/5) survived the 8,700 CFU challenge and 80% (4/5) survived the 87,000 CFU challenge. *Slc11a1*^+^ *Tlr2/4/9^−^*^/^*^−^*, and *Slc11a1*^±^ *Tlr2^−^*^/^*^−^Tlr4*^±^ *Tlr9*^±^ had similar survival with all mice surviving the 8,700 CFU challenge and 60% of *Slc11a1*^+^ *Tlr2/4/9^−^*^/^*^−^*, and 100% of *Slc11a1*^±^ *Tlr2^−^*^/^*^−^Tlr4*^±^ *Tlr9*^±^ survived the 87,000 CFU challenge (Figure 9). This resistance to even very high-dose challenges of LVS, even in the absence of TLR2, highlights the strong effect functional Slc11a1 has the outcome of LVS infection.

## Discussion

Slc11a1 has been implicated in a variety of intracellular infections including *Salmonella* and *Mycobacterium* (20). The exact mechanism of Slc11a1 restriction of pathogen replication is unclear, but some work seems to indicate that it is iron restriction outside of the vesicle. Slc11a1 is a transporter of both ferrous iron (Fe^2+^) and manganese (Mn^2+^) (21), both of which are important in ROS production and suggest a mechanism for the effect of Slc11a1 expression. In *Mycobacterium* infections excess iron can overwhelm the Slc11a1 restriction (22–24). It is clear that Slc11a1 restriction is dependent on pathogens passing through a LAMP-1^+^ vesicle, intracellular pathogens that avoid LAMP-1^+^ vesicles such as *Listeria* are not restricted by Slc11a1 (20). *Francisella* is sensitive to iron starvation when cells are treated with bafilomycin A to limit *Francisella* escape into the cytosol, sequestration of iron by Slc11a1 may play a role in the resistance to LVS infection (25, 26). Initial studies in BMDM indicated that LVS was only slightly deficient in invasion of *Slc11a1*^+^ macrophages and supported no discernable growth. By contrast, LVS replicated robustly in B6 BMDM, indicating an early event after entry that restricts growth in *Slc11a1*^+^ BMDM. There is evidence that *Francisella* is able to spread cell to cell through trogocytosis (27), while not specifically examined in our experiments, the fact that LVS is unable to replicate in *Slc11a1*^+^ cells, either *in vivo* or *in vitro*, indicates cell to cell spread does not allow LVS to escape Slc11a1-mediated resistance.

Previous work had indicated that expression of Slc11a1 enhanced LVS proliferation following footpad inoculation (5). In our hands, we saw no significant difference between B6 and *Slc11a1*^+^ mice following footpad infection (Figure 2). One difference in our study design was using a higher dose of LVS in the footpad (10^3^ vs. 10^2^). This increased dose may account for why we were unable to see the small difference that was seen in the study by Kovarova et al. Additionally, the background of the mouse strains is different (B6 vs. B10), which may also contribute to the fact we saw no difference in the footpad infection. Other genes on chromosome 1 that differ between the *Slc11a1* donor in the A/J and the 129 and could be passengers in the congenics. In our experiments they could have epistatic effects that could also contribute to these differences. By contrast, when we challenged mice i.n., *Slc11a1*^+^ mice were significantly more resistant to LVS than B6 mice. Functional *Slc11a1* expression in C3H/HeN and 129/J mice is not sufficient to survive a highly virulent type A (Strain 33) challenge (28, 29). This might be due to differences in either the genetic background of the mice or differences between *Francisella* Strain 33 and LVS. Other host genetic differences among mouse strains remain to be explored. A recent study by Fink et al. examined quantitate trait loci (QTL) following LVS infection (6). They reported that A/J mice, which are *Slc11a1*^+^, are more sensitive to LVS infection as compared to B6 mice. Recombinant inbred (RI) mice between these two strains were tested and QTL analysis performed. Three QTL were found to be significant, one region on chromosome 1 that includes the *Slc11a1* gene, one on chromosome 2, and a large region of chromosome 19. Further analysis demonstrated the QTL on chromosomes 1 and 2 was epistatic. This indicates that the genetic variation on chromosome 2 of A/J mice acts in concert with the *Slc11a1* region on chromosome 1. Our *Slc11a1^+^* mice, being incipient congenics, are highly likely to be same as B6 mice on chromosome 2, lacking whatever element from A/J is interacting with the *Slc11a1* region on chromosome 1. Combined with our data it seems that functional *Slc11a1* expression in a B6 background in beneficial for LVS infection, whereas with the interactions from chromosome 2 *Slc11a1* expression can be detrimental. Further RI strains, with more founder strains such as the Collaborative Cross, will be needed to narrow the region on chromosome 2 that interacts with *Slc11a1* (30). While the B6 *Slc11a1*^+^ strain does still contain 129 DNA surrounding the *Slc11a1* allele (10), the F1 cross (B6-*Tlr2^−^*^/^*^−^Slc11a1*^D/D^ X B6-*Tlr2,4,9^−^*^/^*^−^Slc11a1*^G/G^) we have made should be heterozygous in that region of chromosome 1. Since this F1 strain behaves similar to the parental strain B6-*Tlr2,4,9^−^*^/^*^−^Slc11a1*^G/G^ we feel confident that the remaining 129 DNA is not confounding our results (Figure 9A). In our hands, all *Slc11a1*^+^ mice showed marked resistance to intranasal LVS infections. *Slc11a1*^+^ mice had little to no detectable bacteria by day 3 post-infection. Additionally, when *Slc11a1*^+^ were challenged i.n. with escalating doses of LVS they were able to survive challenges of 87,000 CFU (80 LD_50_). B6-*Slc11a1*^+^ were also resistant to a fully virulent murine *Francisella novicida* challenge by the intranasal route (data not shown). The *Slc11a1*^+^ mice also had no change in total lung cellularity. B6 mice show a large influx of macrophages and neutrophils as well as increased production of IFN-γ, MCP-1, and IL-6. This lack of change in cellularity may be linked with the lack of cytokine production in *Slc11a1*^+^ mice. Taken together it would seem that resident lung cells are carrying out the rapid clearance of *Francisella*. Previous work by our laboratory and others has demonstrated that *Francisella* can infect a variety of cells and these infected cells differ by route of infection (17, 31–33). Particularly of interest is the fact that alveolar macrophages are the primary infected cell in the lung after intranasal infection whereas after intradermal infection the primary infected lung cells are interstitial macrophages and neutrophils (7). Experiments on PMNs, which do not express *Slc11a1*, indicate that LVS infection of PMNs prolongs their life span, whereas LVS has been shown to induce cell death in macrophages (34–37). These differential effects depending on cell type may help explain the differences seen in *Slc11a1*^+^ mice depending on the route of infection.

The rescue of the TLR2 defect was surprising. TLR2 has been reported by a variety of groups to be critical in survival of *Francisella* infection (8, 11, 12, 15, 38). This work has been carried out in the *Slc11a1^−^* B6 background. Functional *Slc11a1* can not only overcome a lack of TLR2, but *Slc11a1*^+^ *Tlr2^−^*^/^*^−^* mice are more resistant to intranasal LVS infection than B6 mice. B6-*Tlr2/4/*9*^−^*^/^*^−^* (*Slc11a1^−^*) mice are not available to used as a control, though we expect they would behave like B6-*Tlr2^−^*^/^*^−^* mice as TLR4 and TLR9 have little role in *Francisella* infection (39, 40). Further work is needed to determine if *Slc11a1*^+^ mice are resistant to fully virulent human *Francisella* strains such as SchuS4. A variety of live attenuated mutants have been generated in both LVS and the type A strain SchuS4. While attenuation has been relatively easily achieved, full protection against a type A challenge has been rare and even successful vaccines only have protected against limited doses of highly virulent bacteria [reviewed in Ref. (41)]. Since most humans express a functional Slc11a1, retesting of these vaccine candidates in a *Slc11a1*^+^ background may yield more applicable results.

## Author Contributions

DP designed and carried out the experiments. JF and DP analyzed the data and wrote the manuscript.

## Conflict of Interest Statement

The authors declare that the research was conducted in the absence of any commercial or financial relationships that could be construed as a potential conflict of interest.
