# Supplementary figures and images for: Efficacy of Resistance to Francisella Imparted by ITY/NRAMP/SLC11A1 Depends on Route of Infection
Source: Front Immunol. 2017 Mar 15;8:206. doi: 10.3389/fimmu.2017.00206 (PMC5350118; doi:10.3389/fimmu.2017.00206)

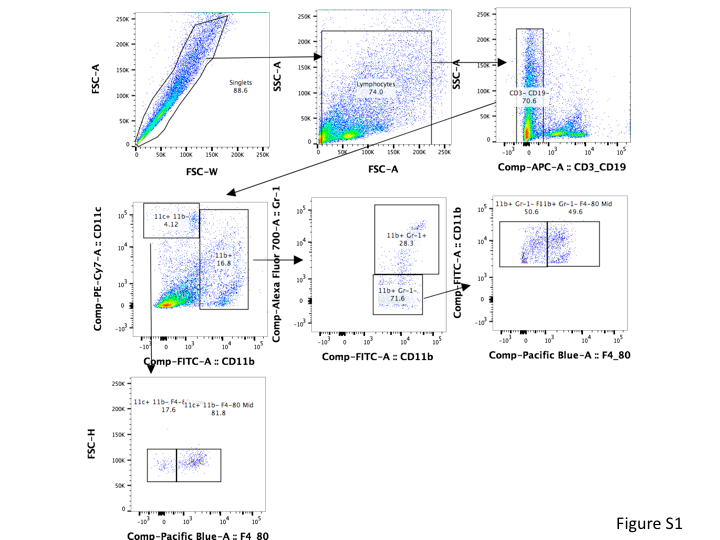

Supplement: Supplementary file 1 [file Image_1.TIFF]
